# A Multi-Institutional Collaborative To Assess the Knowledge and Skills of Medicine-Pediatrics Residents in Health Care Transition

**DOI:** 10.7759/cureus.20327

**Published:** 2021-12-10

**Authors:** Colby Feeney, Emily Hotez, Lori Wan, Laura Bishop, Jason Timmerman, Madeline Haley, Alice Kuo, Priyanka Fernandes

**Affiliations:** 1 Medicine and Pediatrics, Duke University School of Medicine, Durham, USA; 2 Internal Medicine, David Geffen School of Medicine, University of California Los Angeles, Los Angeles, USA; 3 Medicine and Pediatrics, University of California San Diego, San Diego, USA; 4 Medicine and Pediatrics, University of Louisville School of Medicine, Louisville, USA; 5 Internal Medicine and Pediatrics, David Geffen School of Medicine, University of California Los Angeles, Los Angeles, USA

**Keywords:** internal medicine & pediatrics, adolescent and young adult, curriculum, young adult, health care transition

## Abstract

Background

Pediatric to adult health care transition (HCT) is an essential process in the care of youth with special health care needs (YSHCN). Many internal medicine-pediatrics (med-peds) residency programs have developed curricula to teach transition knowledge and skills for the care of YSHCN.

Objective

Using a national med-peds program director quality improvement collaborative to improve transition curriculum, we aim to identify curricular content areas of improvement by describing baseline trainee knowledge and skills taught through existing transition curricula in med-peds programs.

Methods

We analyzed data collected during the 2018-2019 national med-peds program director quality improvement collaborative to improve transition curriculum. Program directors assessed their programs, and trainees assessed themselves on five transition goals by completing a Likert-scale questionnaire. In addition, trainees received an objective assessment of their knowledge through a multiple-choice questionnaire (MCQ).

Results

All 19 programs in the collaborative, and 193 of 316 trainees from these programs, completed the questionnaires. Most programs were based at academic centers (68%) and provided transition training via didactics (63%) and/or subspecialty rotations (58%). More programs had high confidence (95%) than trainees (58%) in goal 1 (knowledge and skills of the issues around transition), whereas more trainees had high confidence (60%) than programs (47%) in goal 2 (understanding the developmental and psychosocial aspects of transition). Programs and trainees self-assessed lower in goals related to health insurance, educational and vocational needs, and application of health care system knowledge to the practice environment (goals 3, 4, and 5, respectively).

Conclusions

Using the assessments of the program directors and resident trainees, we identified subject areas for improvement of transition curricula, including health insurance and the application of health care system knowledge to the practice environment.

## Introduction

The entrance to adulthood can be a perilous time for all patients, especially for those with special health care needs. The young adult years are often marked with complex changes to social structure and needs, declining health, and increased acute care utilization [1-6]. Therefore, the transfer to adult care must be handled with great attention. Health care transition (HCT) requires years of preparation by pediatricians, a planned handoff, and a deliberate intake and integration into adult care by adult providers. Despite approximately 750,000 youth with special health care needs (YSHCN) entering adulthood each year, only 17% discussed transition planning from pediatric to adult medicine with their health care providers [7,8]. Moreover, trainees in pediatric, non-pediatric, and combined internal medicine and pediatrics (med-peds) residency programs lack confidence in transitioning young adults with over 40% of trainees at the end of their training feeling unprepared to perform transition activities [9].

Physician training programs are tasked with providing the education and experiences necessary to teach physicians how to facilitate the transition for their patients. A framework for resident trainee competencies has been described, which accounts for the numerous and complex needs during transition to adulthood [10]. The Health Care Transition Residency Curriculum Collaborative Improvement Network (HCT-CoIN) was formed in 2018 and used this framework to bring together program directors of med-peds residency programs across the country to work on improving the HCT curricula through iterative learning process. 

Our study aims to identify areas of curricular improvement by analyzing the results of program director and resident assessments of knowledge and skills of pre-defined HCT topics.

## Materials and methods

Study design and sample

This is a descriptive study of the baseline data collected as part of a prospective quality improvement learning collaborative. The results of the learning collaborative have not been published. The HCT-CoIN emerged from the larger University of California, Los Angeles (UCLA) Health Care Transition Research Network, funded by the Maternal and Child Health Bureau of the Health Research Services Administration. The UCLA med-peds residency program served as the coordinating and administrative site for all study procedures. The purpose of the HCT-CoIN was to create a toolbox of resources for residency program directors to implement or enhance HCT curricula at their own institutions from June 2018 to February 2019. An incentive of $1000 was provided to all program directors who agreed to participate in the HCT-CoIN. They had the discretion to use the incentive in any way to improve participation in and/or success of the study. Program directors from 22 med-peds residency programs across the country elected to participate in the collaborative at the end of the 2018 Medicine-Pediatrics Program Directors Association (MPPDA) meeting. Three programs withdrew prior to data collection. Data from 19 programs are included in the current analysis. Programs were diverse in geographic location.

Data collection and measures

**Figure 1:**
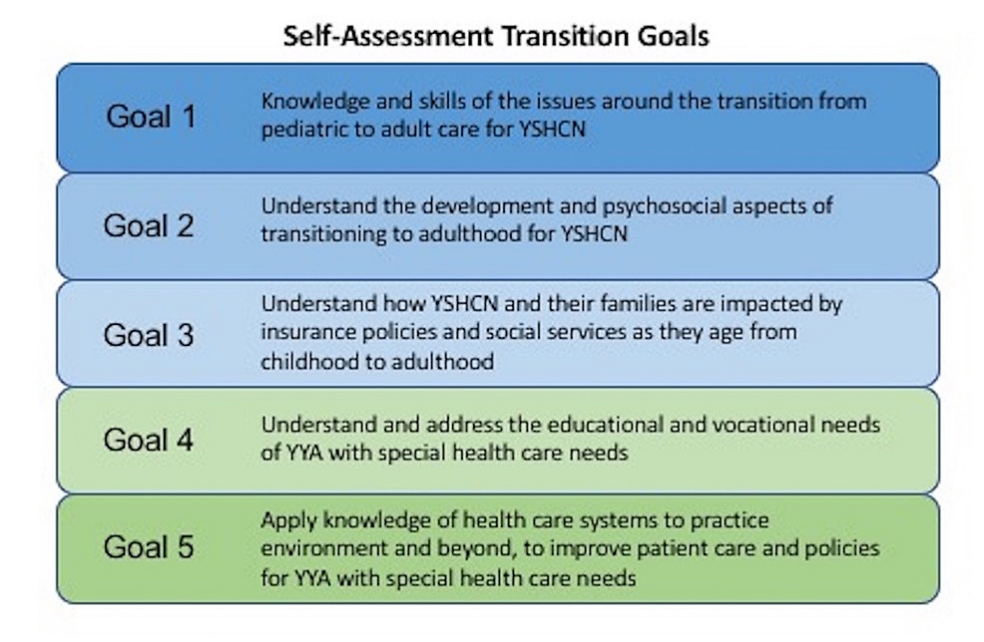
Self-assessment transition goals which were reported by program directors and trainees. YSHCN: youth with special health care needs; YYA: youth and young adults

We analyzed data collected during the 2018-2019 national med-peds program director quality improvement collaborative to improve transition curriculum. Study measures and data collection tools for the HCT-CoIN were developed by the project director (a med-peds residency program director), the quality improvement consultant (a preventive medicine physician), and the project coordinator of the study team at the coordinating site, UCLA. Data collection tools including the self-assessment surveys and multiple-choice questions (MCQ) were designed by the aforementioned study team. The trainee self-assessment and MCQs are provided in the Appendix. The main outcome measure for the current study was an assessment score with regards to five transition goals outlined previously using a modified Delphi model, and this was collected prior to the initiation of the activities of the improvement collaborative [10]. The transition goals are described in Figure 1. Baseline data were collected from the program directors at each of the participating sites. They were surveyed about the setting and size of their residency program and their assessment of their residents’ competency in the five goals of transition care on a scale of 1 to 5. The scale was similar to the scale used by the Accreditation Council for Graduate Medical Education (ACGME) Milestones wherein a higher score reflected greater preparedness or skill for each transition goal [11]. Surveys were distributed electronically via email to program directors and responses were obtained between April and September 2018.

The second source of baseline data was collected from resident trainees in the 19 programs. The resident trainee survey consisted of a self-assessment of their current knowledge of transition care and 15 MCQs created by the study team as no other validated survey was available or applicable to the study. The MCQs were mapped to the transition goals used in the program director and resident self-assessments. MCQs that mapped to more than one goal were questions 1 (goals 1 and 5), 6 (goals 2 and 4), 13 (goals 1 and 3), 14 (goals 2 and 5), and 15 (goals 1 and 5). Each MCQ had one right answer as determined by the coordinating site expert panel. The self-assessment for resident trainees used a Likert scale, similar to the program director assessment, where trainees rated their preparedness on each of the objectives within the five goals of transition care on a scale of 1 to 4. A higher score reflected greater preparedness for the goal. Eighteen of the 19 programs administered the online version of the survey to all their current resident trainees. One program administered a paper-version of the survey to their trainees to improve the response rate. Survey responses were received over the period of June 2018 to October 2018, and those that did not have at least one self-assessment or question answered were excluded from the final analysis.

Statistical analysis

For the purpose of this study, assessment data from program directors and resident trainees were recoded to a binary variable of low-confidence and high-confidence. For program directors, assessment scores of 1-2 were categorized as low confidence, and scores of 3-5 were categorized as high confidence. This mimicked the ACGME Milestones grading in which a resident at a level 3 suggests competency, and anything higher suggests greater proficiency in the Milestone [12]. For resident trainees, self-assessment scores within each goal were obtained by averaging the scores from all objectives within a goal. While the Likert scale for each objective had four distinct categories of “strongly disagree,” “disagree,” “agree,” and “strongly agree,” because we averaged scores on all objectives within a goal, there were scores between 2 (disagree) and 3 (agree) that needed to be accounted for on our analysis. The scores were therefore recorded such that scores of 1-2.5 were assigned as low confidence, and scores of 2.6-4 were categorized as high confidence. We use descriptive and frequency statistics to describe the sample populations. Data from the MCQs are presented by the goals they represent. If a goal was assessed in more than one question, the average correct responses were calculated and presented for each goal. Lastly, we compared the confidence rating in specific goals between program directors and resident trainees, to determine areas of concordance and discordance. 

The host institution and all participating programs received an exemption from their individual institutional review boards.

## Results

Response rates

We received responses from all 19 program directors (100% response rate) which represent 24% of the total med-peds programs in the US in 2018-2019. We received 231 responses from a total of 316 resident trainees (73% response rate). Only 193 were included in the final analysis (61% response rate for completed surveys), because 38 responses were incomplete in that none of the self-assessment questions or the MCQs were answered.

Program and trainee characteristics

Table 1 presents program and trainee characteristics. More than two-thirds of residency programs were based in academic settings (68%), with fewer programs being community-based (37%) or in private clinics (16%). Some programs have clinical training experiences in multiple settings (i.e. inpatient academic hospital-based services as well as community-based clinics). Programs served a variety of population demographics including 84% in urban communities, 53% suburban, and 16% rural communities. The HCT curriculum offerings varied with 63% of programs having a didactic curriculum, 37% providing a required clinic-based experience, 11% with an optional clinic-based experience, and 11% utilizing online modules. More than half (58%) of programs delivered the HCT curriculum through subspecialty rotations. Trainee participation was evenly distributed by year of training: post-graduate year PGY1 28%, PGY2 21%, PGY3 27%, PGY4 25%. 

**Table 1:** Program and trainee characteristics

Program characteristics	N (%) (n = 19)
Type of curriculum	
Didactics	12 (63.2%)
Clinic-based (required)	7 (36.8%)
Clinic-based (elective)	2 (10.5%)
Online modules	2 (10.5%)
Sub-specialty rotation	11 (57.9%)
Clinical setting	
Academic	13 (68.4)
Community-based	7 (36.8%)
Private clinic	3 (15.8%)
Population demographic	
Urban	16 (84.2%)
Suburban	10 (52.6%)
Rural	3 (15.8%)
Additional program characteristics	Median (Range)
Number of residents per program	16 (8, 24)
Number of core faculty	5 (1, 16)
Trainee characteristics	N (%) (n = 193)
Trainees by post-graduate year (PGY) level	
PGY1	53 (27.5%)
PGY2	40 (20.7%)
PGY3	52 (26.9%)
PGY4	48 (24.9%)

Multiple-choice question responses by goal

The percentage of resident trainees with correct responses to each MCQ can be found in Table 2. The median number of correct MCQs was 12/15 (Range: 5 -15). The majority of questions had more than 80% correct responses, with the exception of questions 1 (55% correct), 8 (72% correct), 9 (37% correct), 14 (45% correct), and 15 (62% correct). Resident trainees on average scored better in goals 2 and 4 (81% and 91% correct responses respectively) compared to goals 1, 3, and 5 (69%, 73%, and 61% correct responses respectively). 

**Table 2:** Trainee responses to multiple choice questions (MCQs). YSHCN: Youth with special health care needs; YYA: Youth and young adults

Individual MCQs by goal	N (%)
Goal 1- Knowledge and skills of the issues around the transition from pediatric to adult care for YSHCN	
Q1	107 (55.4%)
Q13	173 (89.6%)
Q15	120 (62.2%)
Average correct responses	133.3 (69.1%)
Goal 2- Understanding the development and psychosocial aspects of transitioning to adulthood for YSHCN	
Q3	155 (80.3%)
Q4	177 (91.7%)
Q5	177 (91.7%)
Q6	183 (94.8%)
Q7	160 (82.9%)
Q8	138 (71.5%)
Q12	172 (89.1%)
Q14	87 (45.1%)
Average correct responses	156.1 (80.9%)
Goal 3- Understanding how YSHCN and their families are impacted by insurance policies and social services as they age from childhood to adulthood	
Q9	72 (37.3%)
Q10	178 (92.2%)
Q13	173 (89.6%)
Average correct responses	141 (73.1%)
Goal 4- Understanding and addressing the educational and vocational needs of YYA with special health care needs	
Q6	183 (94.8%)
Q11	169 (87.6%)
Average correct responses	176 (91.2%)
Goal 5- Applying knowledge of health care systems to practice environment and beyond, to improve patient care and policies for YYA with special health care needs	
Q1	107 (55.4%)
Q2	154 (79.8%)
Q14	87 (45.1%)
Q15	120 (62.2%)
Average correct responses	117 (60.6%)
Total MCQs	Median (Range)
Total MCQ correct	12.0 (5, 15)

Self-assessment responses by goal

**Figure 2:**
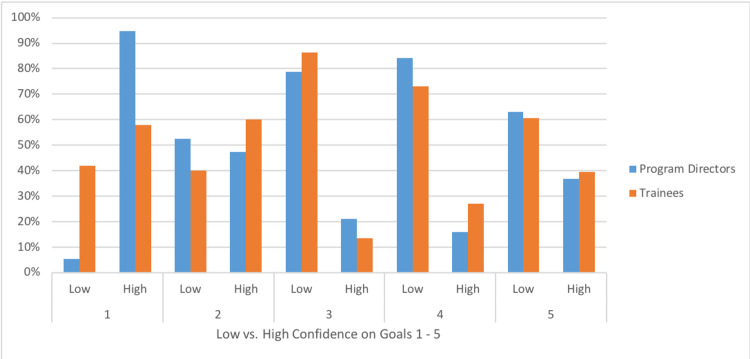
Program Directors’ vs. Trainees’ Self-Assessment of Confidence on Transition Goals Program Directors (n = 19); Trainees (n = 193). Goal 1- Knowledge and skills of the issues around the transition from pediatric to adult care for youth with special health care needs. Goal 2- Understanding the development and psychosocial aspects of transitioning to adulthood for youth with special health care needs. Goal 3- Understanding how youth with special health care needs and their families are impacted by insurance policies and social services as they age from childhood to adulthood. Goal 4- Understanding and addressing the educational and vocational needs of youth and young adult with special health care needs. Goal 5- Applying knowledge of health care systems to practice environment and beyond, to improve patient care and policies for youth and young adults with special health care needs.

Figure 2 presents frequencies and percentages for program director assessments vs. trainee self-assessments of goals. For goal 1, most program directors (95%) reported high confidence that their trainees met the goal. More than half of the trainees also reported high confidence (58%), while 42% reported low confidence. For goal 2, there was near even distribution of low confidence (53%) versus high confidence (47%) in the program directors’ self-assessment. This distribution differed from the trainee assessment with 60% reporting high confidence and only 40% reporting low confidence. With respect to goal 3, only 21% of programs reported high confidence, which was similar to the proportion of trainees reporting high confidence (14%). For goal 4, more programs (84%) reported low confidence than reported in any other HCT goal. Similarly, 73% of trainees rated themselves as low confidence. Lastly, the majority of programs reported low confidence in goal 5 (63%), with a similar proportion of trainees reporting low confidence (61%).

Trainee self-assessments were also analyzed by post-graduate year level. For all five goals, a larger proportion of trainees had high confidence in each subsequent training level (Table 3).

**Table 3:** Learner self-assessment by post-graduate year (PGY) level

Goal/PGY	Low Confidence		High Confidence		Total
	n	%	n	%	
Goal 1					
1	32	60.38%	21	39.62%	53
2	22	55.00%	18	45.00%	40
3	19	36.54%	33	63.46%	52
4	8	16.67%	40	83.33%	48
Goal 2					
1	33	62.26%	20	37.74%	53
2	19	47.50%	21	52.50%	40
3	16	30.77%	36	69.23%	52
4	9	18.75%	39	81.25%	48
Goal 3					
1	51	96.23%	2	3.77%	53
2	35	87.50%	5	12.50%	40
3	41	78.85%	11	21.15%	52
4	40	83.33%	8	16.67%	48
Goal 4					
1	40	75.47%	13	24.53%	53
2	32	80.00%	8	20.00%	40
3	36	69.23%	16	30.77%	52
4	33	68.75%	15	31.25%	48
Goal 5					
1	41	77.36%	12	22.64%	53
2	26	65.00%	14	35.00%	40
3	32	61.54%	20	38.46%	52
4	18	37.50%	30	62.50%	48

## Discussion

Program directors and residents differed in their self-assessed confidence of transition skills and knowledge. Understanding how YSHCN and their families are impacted by insurance policies and social services as they age (goal 3) and improving patient care and policies for youth and young adults (YYA) with special health care needs by improving the application of knowledge of health care systems to practice environment and beyond (goal 5) are two subject areas of residency transition curriculum needing improvement, as evidenced by low self-assessment scores by program directors and residents.

There is a growing literature showing the importance of transition to adult care within specific disease conditions [13-15]. Ours is one of the few studies which identifies specific transition care topics in need of further curricular development from the perspectives of both residency program directors and resident trainees. Previous literature has shown that resident trainees prefer learning about pediatric-to-adult transitions through multiple modalities, with a predominant emphasis on case-based or experiential learning. Research also supports longitudinal curricula throughout training and a multi-modal approach. Trainee self-confidence in transition skills can be increased by exposure to YSHCN, role-modeling of transition skills, and increased experience and practice [9,16].

Previous studies have not evaluated both self-assessment and objective measurement of knowledge related to transition goals. Our study showed discordance between resident self-assessment and objective measurement of knowledge related to transition goals. The discordance was most pronounced for goal 4 (understanding and addressing the educational and vocational needs of YYA with special health care needs), in which residents self-assessed as having low confidence, though most residents correctly answered the MCQs related to the goal. This observation may suggest that the teaching modalities utilized by many combined med-peds residency training programs are effective in teaching knowledge regarding HCT for YSHCN. Transition-related curricula variably consist of didactic teaching and experiential learning in a variety of settings (adolescent-specific rotations, continuity clinic, sub-specialty rotations, etc.). There may be a difference in the perceived mastery of transition care that is learned outside of a defined transition-specific experience or that is only presented in traditional didactic methods. Graduate medical educators could incorporate multiple modalities of transition curriculum delivery throughout residency and highlight informal curricula where it exists to ensure concordance between subjective and objective assessments of resident trainees.

There were several limitations to our study. Programs and residents of only one specialty (combined medicine-pediatrics) were surveyed and did not include other primary care specialties that play a role in HCT such as pediatrics, internal medicine, and family medicine. The sample was biased towards med-peds programs with pre-existing HCT curricula or plans to develop one. Although the transition goals were the same, the program self-assessment scale was slightly different than the resident self-assessment, making comparisons between the two groups not ideal. We addressed this limitation by grouping the self-assessments into high and low confidence categories rather than comparing the raw scales.

The differences between the self-assessments of program directors and resident trainees for transition goals may suggest problems with results validity. However, as medical educators, we believe those differences to be true. The differences in self-assessments may be from a lack of standard competencies on which to assess learners in their transition knowledge and skills. Competency-based medical education (CBME) centers on education and training using standard practices across training programs. Up to this point, many med-peds residency programs have created and implemented their own curriculum to teach HCT utilizing various teaching modalities including didactics, online modules, electronic health record tools, small group discussions, case studies, pediatric and internal medicine dyads, or direct patient care activities [17-19]. However, there is a lack of standardization of educational content and assessment in teaching HCT to residents. 

Studies have shown that ACGME milestones have played an important role in identifying holes in existing curricula, actively engaging resident trainees in their own learning, and assessing the progress of trainees over time [20-22]. Indeed, the milestones are not intended to encompass all curricula necessary for training physicians to be ready for independent practice. However, one solution to standardize the educational content of HCT would be to develop specific sub-competencies (milestones) within the appropriate six core competencies that would address the core elements of HCTs. Such milestones would inform curricular content and allow the use of established assessment methods to determine competence in HCT [23]. These standardized milestones would be valuable to guide competency in HCT not only in the specialty of combined medicine-pediatrics but also in other primary care specialties such as family medicine, pediatrics, and internal medicine.

## Conclusions

Optimal health care for YSHCN depends on high-quality transition, which requires education and training. Having clear goals and objectives at the outset of curricular delivery, preferably adopted from nationally recognized ones, is important to ensure alignment between program directors’ and trainees’ expectations. Future research should be done on assessment methods of transition skills and knowledge beyond self-assessments, ideally those that could be applied within clinical practice to directly observe activities that support comprehensive HCT. This study provides assessment data of med-peds trainee knowledge and skill acquisition from the existing transition curriculum and reveals that further instruction is needed regarding health insurance and the application of health care system knowledge in order to provide quality transitional care to YSHCN.

## Appendices

Transition care residency curriculum project survey

As part of a survey of training on transition care, please complete the following questionnaire. Your answers are entirely anonymous. This information WILL NOT be used for personal evaluation. Do not record your name anywhere on the sheet. If this information is ever published or disseminated, we may identify the institution but not the individual.

We estimate the total completion time of the survey to be 15-20 minutes. Thank you in advance for your participation.

General Information

Unique ID Code

Which best describes you?

Which year of training are you in?

__________________________________ (Please enter in order: (1) the first letter of your middle name, (2) the first letter of the month you were born, (3) the last digit of your SSN, (4) the first letter of the city you were born in.)

Pediatric Resident Internal Medicine Resident Medicine-Pediatrics Resident Family Medicine Resident Other

__________________________________

PGY1 PGY2 PGY3 PGY4 Other

__________________________________

What is the name of your residency training program?

Beaumont Health Brigham and Women's Hospital/Boston Children's Hospital The Johns Hopkins Hospital Loyola University Medical Center Medical University of South Carolina Medical College of Wisconsin Spectrum Health Stony Brook University University of California, San Diego University of Colorado University of Louisville University of Missouri-Kansas City University of Nebraska Medical Center Virginia Commonwealth University Health Brown University Detroit Medical Center/Wayne State University Duke University Geisinger Medical Center PennState Health University of Illinois, Chicago University of Texas, Southwestern Other

__________________________________

Transition Goals and Objectives: How much do you agree or disagree with the following statements?

Goal 1: Knowledge and skills of the issues around the transition from pediatric to adult care for youth with special healthcare needs (YSHCN)

1.  I understand the differences between adult and pediatric care, including cultural and practice differences, systems of support offered to patients and families.

2.  I understand the elements of transition preparation (i.e., key elements of patient, family, and clinician preparation, as well as timing of transition preparation) in the scope of adolescent and young adult practice.

3.  I can work as an effective member of an inter-professional team (including parents, family, primary care providers, and specialists) to support youth with health care transitions.

4.  I can apply effective coding and billing practice for transition care.

Strongly disagree

Disagree

Agree

Strongly agree

Goal 2: Understanding the development and psychosocial aspects of transitioning to adulthood for YSHCN

1.  I can recognize and describe developmental considerations in typical adolescence and emerging adulthood, and the delivery of developmentally appropriate care.

2.  I can identify and address the mental health needs in youth/young adults (YYA) with special health care needs.

3.  I can identify and manage reproductive and sexual health issues in YYA with special health care needs.

4.  I can provide effective counseling to increase adherence to medical recommendations for YYA and their families.

5.  I can collaborate with YYA and families to develop advance care plans when appropriate, including advance directives and identifying goals of care.

6.  I can perform and document a psychosocial assessment of a YYA with consideration of confidentiality issues.

7.  I understand the medical-legal and financial aspects of care for YA with developmental disabilities (e.g., guardianship and conservatorship).

Strongly disagree

Disagree

Agree

Strongly agree

Goal 3: Understanding how YSHCN and their families are impacted by insurance policies and social services as they age from childhood to adulthood

1.  I can identify key resources for patients and families in the home environment including home care services and durable medical equipment (DME).

2.  I understand insurance types and eligibility and how changes to insurance status and availability across the pediatric to adult transition impacts access to care.

3. I can identify available community resources for housing and residential services for YYA with developmental disabilities.

4.  I can identify government income support programs at the local, state, and national levels for YYA with special health care needs.

Strongly disagree

Disagree

Agree

Strongly agree

Goal 4: Understanding and addressing the educational and vocational needs of YYA with special health care needs

1.  I understand the rights granted under the Individuals with Disabilities Education Act and Section 504 of the Rehabilitation Act.

2  I can collaborate with youth and families to help them access education resources needed to maximize educational attainment, including requesting school accommodations and participating in Individualized Education Plan (IEPs).

Strongly disagree

Disagree

Agree

Strongly agree

Goal 5: Applying knowledge of health care systems to practice environment and beyond, to improve patient care and policies for YYA with special health care needs.

1.  I can identify and implement appropriate transition-related policies.

2.  I can advocate for the needs of YYA with special health care needs.

3.  I can conduct regular transition readiness assessments and create prioritized actions for youth development.

4.  I can utilize a transfer package, including readiness assessment, plan of care, medical summary and emergency care plan, and if necessary, legal documents, condition fact sheet, and additional provider records.

Strongly disagree

Disagree

Agree

Strongly agree

Multiple choice questions

1.  Maria is an 18-year-old female with focal segmental glomerulosclerosis. She is a senior in high school and was just accepted into a nursing program at the local university. From a health care transition perspective, what is the best clinician scenario for her to have?

She should keep her pediatrician and pediatric specialists until she graduates from college

She should transfer to an adult primary care provider but keep her pediatric specialists

She should transfer to adult primary care and specialty providers

She should rely on student health because in college she should focus on her studies

2.  A local children's hospital has just completed an internal audit of its patient outcomes and identified that the health utilization of 18 to 25-year-old young adults includes fewer visits to primary care and more visits to the Emergency Department. What is the first step that the institution should take to address this issue?

Develop a policy on how pediatric patients should connect with adult providers and services

Develop a registry to track young adults with special health care needs

Develop resources to help young adults keep their health insurance

Have social workers available to address mental health issues in young adults

3.  A 17-year-old male was diagnosed with type 1 diabetes when he was five years old and has been on insulin ever since. His mother still administers his shots, refills his medications, and makes his doctors' appointments. At what age should he be doing these tasks himself?

When he becomes an adult at age 18 years

When he started high school

When he started middle school

When he demonstrated the ability to be responsible for any self-care task

4.  A 22-year-old obese male with Prader-Willi syndrome is extremely fearful of coming to the doctor's office for any visit. He was diagnosed with type 2 diabetes at age 15 years and has been lost to follow-up from the office for the past five years. He has had no labs in the interim and his parents report he needs his tetanus shot as well. He is minimally verbal, weighs 320 pounds, and flails and screams when he is restrained. What is the best way to get blood for lab tests?

Have four individuals restrain him while a nurse draws the blood and delivers the shot

Send him to the ER to have the blood drawn and shot given under conscious sedation

Coordinate the blood drawn and vaccine with another procedure such as a dental cleaning or ear wax removal under anesthesia

Treat the diabetes without drawing labs

5.  A 24-year-old woman with cystic fibrosis (CF) reports being in a steady and loving relationship with her fiancé. They are planning to be married within the next year and would like to start a family within five years. Her primary care provider should:

Refer her to reproductive endocrinology because women with CF cannot get pregnant without reproductive technology assistance

Provide guidance on birth control because CF-pregnancies are considered "high risk" and she should not risk her own health by having children

Refer to an obstetrician-gynecologist because CF is a specialized health condition that might affect her chances of getting pregnant

Counsel her that she is unlikely to get pregnant and to consider adoption if she wants to have children

6  An 18-year-old with learning disabilities and attention deficit hyperactivity disorder (ADHD) is about to exit the K-12 system. He is not college-bound but is interested in learning about automotive mechanics. His parent is concerned about staying involved and informed about his health. You should:

Advise the parent to apply for conservatorship so that they can make all health care and legal decisions for their child

Advise the parent to create opportunities for him to gain skills to be able to work and live independently in the future

Advise the parent to create a financial plan and trust for their child

Advise the parent that given how well he did in high school, he should aim higher than automotive mechanics

7  A 25-year-old woman with Down syndrome comes to the office stating that she has had difficulty sleeping lately. She reports increased difficulty concentrating and irritability and decreased appetite. Upon further probing, you find out that she has also recently stopped attending her weekly social group shortly after her mother's death approximately six months ago. She says that although she used to enjoy attending that group, she stopped attending because she did not like the new group leader who took over about four months ago. The best course of action is to:

Do nothing, this is a typical grief reaction to her mother's death

Prescribe medication to help her sleep

Refer to behavioral health services for possible depression

Encourage her to find a different social group

8.  Survivorship care planning for a 23-year-old survivor of childhood cancer should always involve:

Behavioral health counseling

Regular neurological exams to monitor for cognitive, motor, and/or sensory deficits

Routine HIV testing

Complete metabolic screen annually

9.  A 26-year-old male with schizophrenia has been stable on medications and has worked since he graduated from college. Over the past nine months, he has developed paranoid hallucinations. Because he thinks the government is trying to "get into his mind", he refuses to go to work and loses his job. At times he will stand on street corners and yell at people walking by. One time he grabbed a woman's arm, thinking she was holding a grenade (it was actually her cell phone). His mother is extremely concerned because she still works and is unable to watch him at home. After connecting the patient with his psychiatrist to adjust his medications, the next step in his care would be:

Conducting a full battery of neuropsychological testing to determine whether he's developed another psychiatric condition

Encourage his mother to apply for Supplemental Security Income (disability)

Tell his mother that it's her responsibility to monitor his behavior so she will need to quit her job

Have him moved to a residential placement because he is now a danger to others

10.  A 25-year-old woman is s/p liver transplant when she was 16 for fulminant hepatic failure from hepatitis A infection. She is currently on her parents' insurance, which is a commercial PPO plan provided through her father's work. Her 26th birthday is in three months. She is currently unemployed and living at home. She continues to have periodic hospitalizations due to complications from her medical condition. What is her best option for continued insurance coverage after she turns 26?

Medicaid through disability (Supplemental security income (SSI))

Have her father pay an additional premium to keep her on his policy

Enroll her in community college because she can get student health insurance

There are no options, she will have to be uninsured and get her care through the ER

11.  A 22-year-old male with autism spectrum disorder and mild intellectual disability has been working in a job program for adults with special health care needs. He reports enjoying his work duties and interacting with his co-workers. However, his guardian expressed concerns that he may be experiencing bullying from his co-workers and that they sometimes seem to take advantage of him by getting him to do their work for them.

The patient's guardian should raise these concerns with his supervisor for assessment and intervention

Provide reassurance and coaching to the guardian to reduce the overprotectiveness

Link patient to vocational rehab agency for replacement in a different position

Apply for SSI because his disability precludes him from working effectively

12.  A 16-year-old female with epilepsy is currently about to start her junior year of high school. Her father states that he has begun the process of bringing up college preparation with his daughter, but he has become significantly frustrated and tired because she often becomes highly defensive and angry during these conversations. While he is supportive and wants to help her develop skills at an appropriately comfortable pace, he is also concerned that her resistance and lack of motivation will result in her not acquiring the life skills that she needs to live independently and she will be dependent on him for life.

Refer the family to the school IEP team because it is their role to help in transition planning

Reassure the father that all teenagers behave in rebellious ways and her current defensiveness and resistance will likely self-resolve over time

Meet with the patient separately to discuss underlying concerns that may be causing her defensiveness and resistance

Tell the father that if she is not ready for college, he should let her take a gap year

13.  Which of the following interventions would not improve adult outcomes for YYA with special health care needs?

Having a dedicated care coordinator focused on optimizing each young adult's transfer from pediatrics to adult care

Using the electronic medical record to facilitate communication between providers

Having information sessions for YYA and their families about how to transition from pediatrics to adult healthcare

Keeping YYA with pediatrics until age 26 because they can stay on their parents' health insurance until then

14.  A 19-year-old female with an intellectual disability who functions at a four-year-old level is brought to the Emergency Department following a motor vehicle accident. Health Insurance Portability and Accountability Act (HIPAA) prohibits providing any information to anyone without a patient's consent. The young woman is distraught but consolable, and after several hours, her parents call the ER demanding information about her condition.

Given the situation, you can confirm her presence in the ER, but give no details about her condition

Without evidence of conservatorship, you tell the parents nothing

Because of her intellectual disability, you tell the parents everything they want to know

Although you understand that you would be violating HIPAA if you tell the parents anything, you give the parents the information they want because the moral aspect of the situation outweighs the legal aspect

15.  A 20-year-old male with congenital heart disease presents to the ER with fever, dyspnea, and chest pain. The decision is made to admit to rule out endocarditis, but there are no pediatric beds available, and the patient has never been admitted to the adult floor before. What should be done for the patient?

Keep him in the ER until a pediatric bed is available

Transfer him to the adult floor with the adult ward team until a pediatric bed becomes available, then transfer him to pediatrics

Transfer him to the adult floor with the adult ward team for the entire admission

Transfer him to the pediatric ICU (PICU) even though his status does not warrant ICU level care
